# Primary neuroendocrine tumor of the liver: A diagnostic dilemma in the management of liver mass in pregnancy

**DOI:** 10.1016/j.radcr.2022.03.056

**Published:** 2022-04-08

**Authors:** Ian Chik, Jane Wai Yee Chuah, Zamri Zuhdi, Firdaus Hayati

**Affiliations:** a Department of Surgery, Faculty of Medicine, Hospital Canselor Tuanku Muhriz, Cheras, Kuala Lumpur, Malaysia; b Department of Surgery, Faculty of Medicine and Health Sciences, Universiti Malaysia Sabah, Kota Kinabalu, Sabah, Malaysia

**Keywords:** Case report, Hepatocellular carcinoma, Neuroendocrine tumor

## Abstract

Neuroendocrine tumor (NET) commonly occurs in the gastrointestinal tract, however primary NET of the liver is rare, especially during pregnancy. We present a 34-year-old pregnant woman gravida 3 para 2 at 16 weeks period of gestation with primary liver NET discovered incidentally during the antenatal check-up. She has no risk factors for hepatocellular carcinoma. Her serum alpha-fetoprotein was elevated. A plain magnetic resonance imaging (MRI) of the liver delineating a large well-defined exophytic liver mass at segment V/VI measuring 7.1 × 7.4 × 7.8 cm. Given inconclusive MRI findings coupled with low-risk factors of HCC, we had decided to follow up her liver mass with imaging 6 weekly. She then underwent a right hepatectomy with a caesarean delivery at 32 weeks of gestation in the same setting. The histopathological formal report revealed a neuroendocrine tumor, grade 2 with a Ki-67 index of 3% with negative lymphovascular and perineural invasion, but positive for porta hepatis lymph nodes metastasis. A follow up after 1 year shows both patient and her infant are healthy. Antenatal discovery of liver masses poses a diagnostic and management dilemma to clinicians. A multidisciplinary approach and collective decision making are crucial to determine the best approach tailored to the maternal and fetal benefit. In cases of inconclusive non-contrast MRI in pregnancy with low-risk factors and lack of clinical evidence of HCC, follow-up with imaging modalities aiming to intervene at the third trimester can offer safer, and promising outcomes.

## Introduction

Neuroendocrine tumor (NET) arises mainly from the gastrointestinal tract (50%-70%) and lung (20%-30%) with a predilection to metastasize to the liver [1,2]. Primary NET of the liver is extremely rare and difficult to diagnose accurately preoperatively if compared to other solid liver masses, especially hepatocellular carcinoma (HCC) [2]. With recent advancements and the availability of ultrasonography worldwide, liver masses despite being rare in pregnancy are increasingly being detected [3]. Although routine antenatal ultrasound does not assess maternal solid organs regularly, pregnant patients may undergo an additional abdominal ultrasound examination during the investigation of abdominal symptoms. Due to a limited choice of radiological investigations in a pregnant patient, the differentiation of a solid liver mass itself becomes even more challenging for clinicians. We report a case of primary liver NET in a pregnant patient that required detailed planning and teamwork between multiple disciplines to ensure the most beneficial outcome for both patient and her unborn fetus.

## Case report

A 34-year-old lady gravida 3 para 2 at 16 weeks of gestation was noted to have worsening creatinine on her renal profile during a routine antenatal follow-up. She was a known case of type 2 diabetes mellitus, hypertension, and Beta thalassemia trait. She works as a school teacher and has neither risk factors for HCC nor a family history of malignancy. During the physical examination, she was not icteric, and without signs of chronic liver disease. There was a gravid uterus which was consistent with a period of gestation but no other masses were palpable. The rest of her general examination was unremarkable. The renal profile revealed a serum creatinine of 105 from 96 umol/L (normal reading: 50-98 umol/L) with a normal serum urea level. The liver function test was normal; however, the tumor marker of serum alpha-fetoprotein was elevated with a reading of 65.5 ng/mL (normal reading: <8.8 ng/mL).

**Fig. 1:**
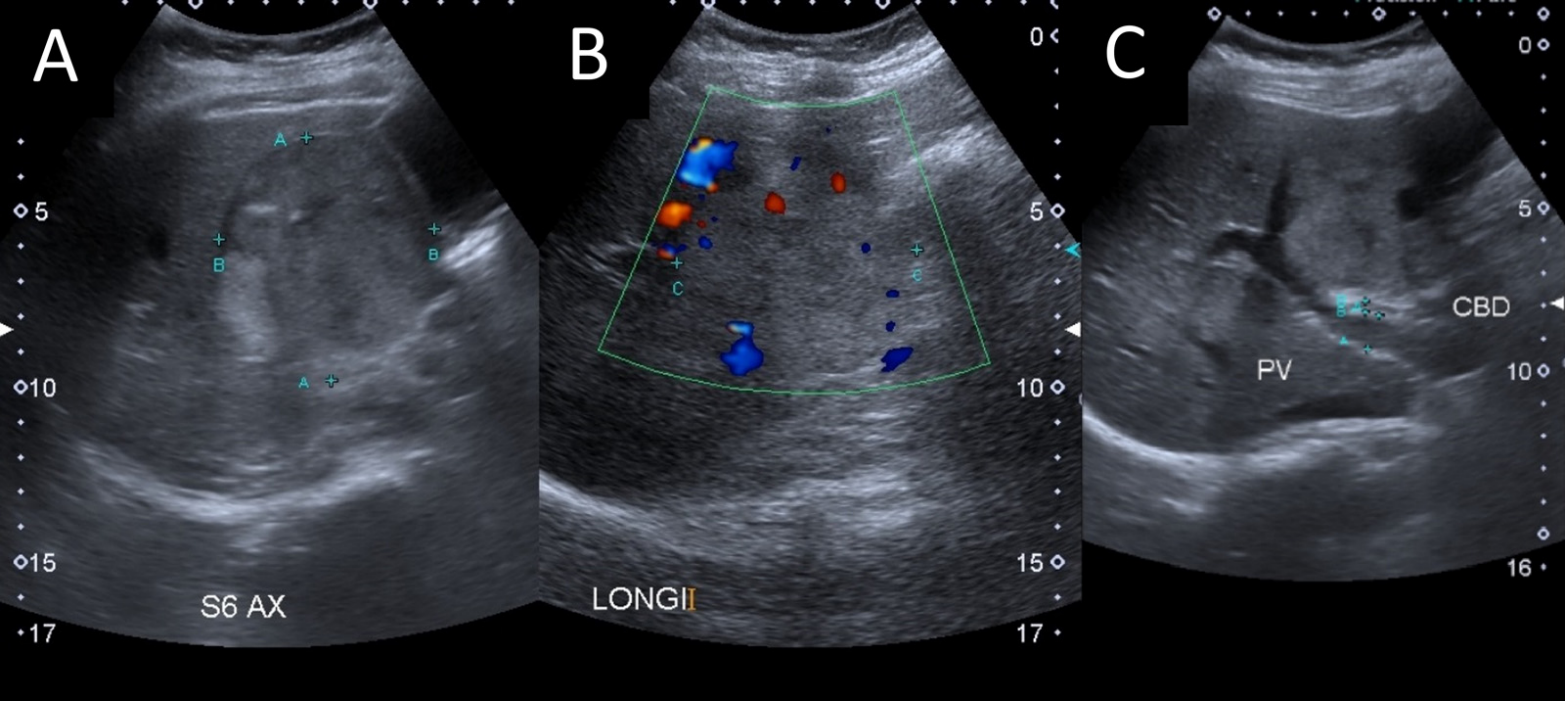
Ultrasound images demonstrating a segment VI liver lesion (A) measuring 6.4 × 6.6 × 7.4cm. The lesion had a focus of calcifications and few cystic lesions. Internal vascularity (B) was seen within. The mass caused effacement of the portal vein and displacement of the gallbladder anteriorly (C).

**Fig. 2:**
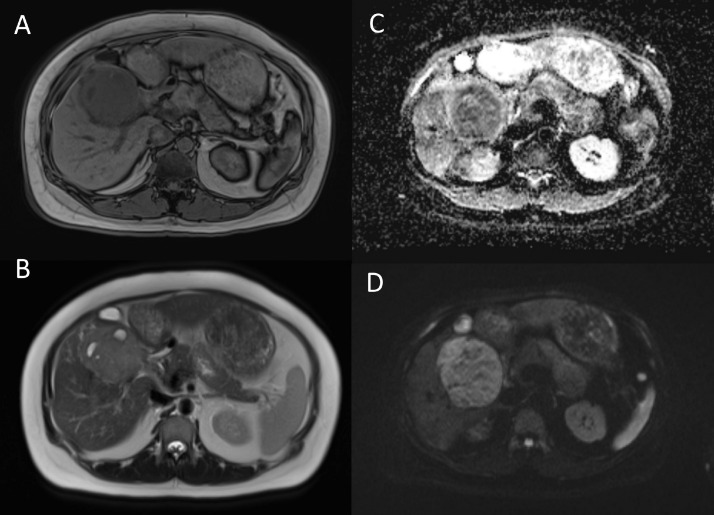
MRI images showing a large well-defined exophytic lesion at segment V/VI 7.1 × 7.4 × 7.8 cm with few cystic components seen within, and a clear fat plane with surrounding structures. On T1WI it has heterogenous hypointense signal (A) and on T2WI a hyperintense signal (B). No signal loss in out-of phase sequence. Evidence of restricted diffusion seen showing high signal on DWI (C) and low signal on ADC sequence (D).

Abdominal ultrasonography was subsequently performed to look at her urinary tract anatomy, however incidentally a large liver lesion was found instead. The liver lesion was noted to be heterogeneous and hyperechoic, measuring 6.4 × 6.6 × 7.4 cm and located at segment VI (Fig. 1). A plain magnetic resonance imaging (MRI) of her liver was then performed, delineating the liver mass to be a large well-defined exophytic lesion at segment V/VI measuring 7.1 × 7.4 × 7.8 cm (Fig. 2) and had a clear fat plane with surrounding structures. There was no biliary tree dilatation and the portal vein showed normal flow void. It also demonstrated heterogeneous hypointense signal on T1W1, and hyperintense signal on T2W1 which was suggestive of HCC although not conclusive in a plain MRI. Few cystic components were seen within the mass. Evidence of restricted diffusion was seen showing a high signal on DW1, and low signal sequence on ADC sequence indicating a highly-cellulated lesion suggestive of malignancy. Although HCC does not commonly occur in non-cirrhotic patients, we could not ignore the malignant characteristics of the liver lesion on MRI, and the fact at least a LI-RADS 3.

**Fig. 3:**
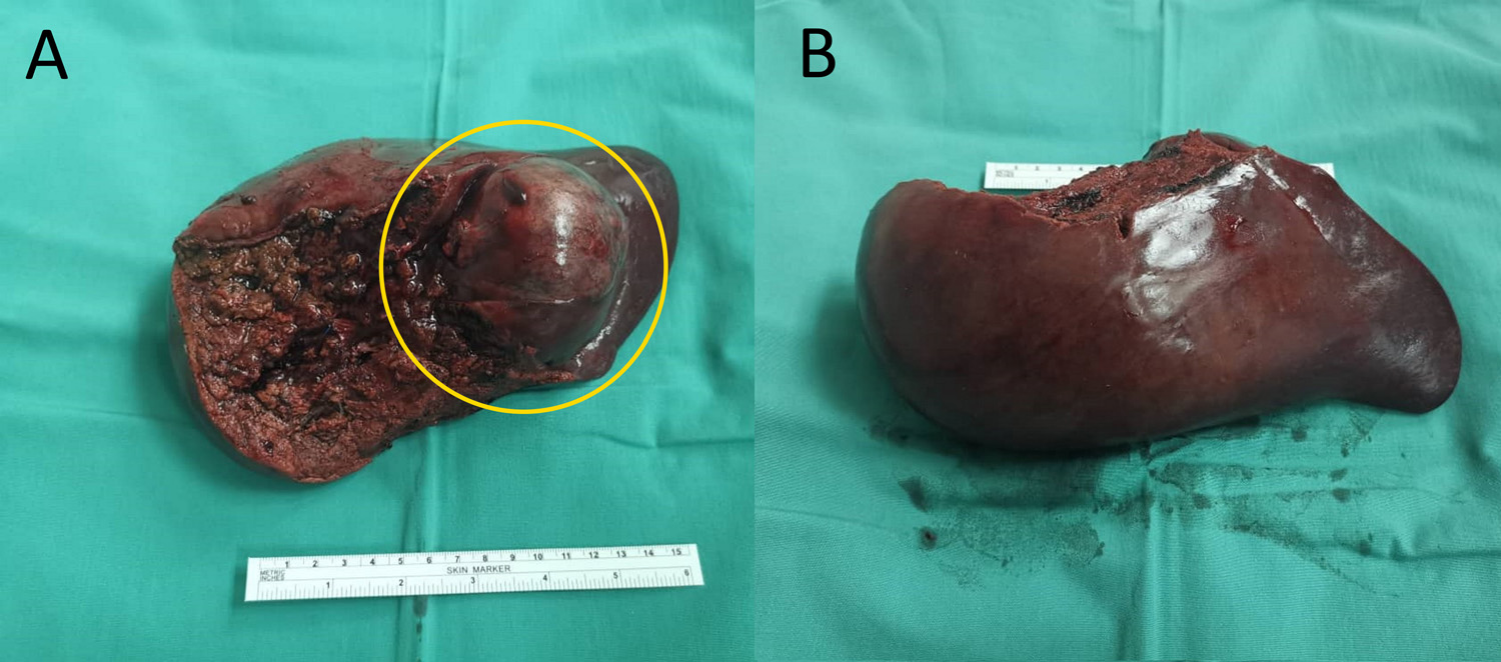
Right hepatectomy specimen at medial (A) and lateral (B) view showing a large, rounded mass (circle) measuring approximately 6 × 6 cm. The liver was not cirrhotic.

Following a discussion between the obstetric, pediatric, hepatobiliary and radiology multidisciplinary meetings, a collective decision was made to perform an elective caesarean delivery at 32 weeks of gestation as well as a right hepatectomy in the same setting considering the inconclusive MRI and feto-maternal wellbeing. MRI liver volumetry was also performed which showed residual liver of 40%. Before the operation, the risks and complications were explained in detail to both patient and her husband. She delivered a baby boy via a standard caesarean section weighing 1700 grams with an Apgar score of 7. The baby was admitted into the neonatal intensive care unit for close monitoring and weight gain. She on the other hand underwent standard right hepatectomy, after an intraoperative ultrasound revealing no other liver lesions. It had compressed the portal vein intraoperatively. The tumor measured 6 × 6 cm, was firm in consistency and had no evidence of liver cirrhosis (Fig. 3). Her post-operative period was uneventful and she was discharged 5 days after. Her son was born prematurely, but healthy post-operatively, and was discharged after 2 weeks with good weight gain.

The histopathological formal report revealed a neuroendocrine tumor (NET), grade 2 with a Ki-67 index of 3%. It was negative for lymphovascular and perineural invasion, but positive for porta hepatis lymph nodes metastasis (1/1). Surgical resection margins and vascular stump were free from tumor involvement. Due to the rarity of a hepatic endocrine neoplasm as a primary instead of a metastatic deposit, a follow-up contrasted computed tomography (CT) scan of thorax, abdomen and pelvis was performed and did not show any recurrence or residual tumor and there were no other findings to suggest a primary neuroendocrine tumor elsewhere. She was well without any postoperative complications upon clinic review a week after she was discharged. A positron emission tomography (PET)-CT Gallium-68 DOTATATE scan was performed and revealed neither abnormal increase in Gallium-68 DOTATATE uptake at the resected liver site nor in any other organs to suggest a primary neuroendocrine tumor site. At her recent 1 year follow up postoperatively, both the patient and her son are healthy and well.

## Discussion

Dealing with a liver mass during pregnancy, it poses not only a diagnostic dilemma but a management challenge to both obstetricians, and surgeons alike. Although most liver masses in pregnancy have been reported to be benign, the diagnosis of exclusion for malignant cancers especially HCC is vital. Previously published articles have reported an increased incidence of tumor rupture and hemorrhage of approximately 10% of HCC found during pregnancy [4]. This could be due to the tumor's growth being accelerated by hormonal and immunologic changes that occur throughout pregnancy. It has been reported that the overall 1-year survival of HCC in pregnancy was only 23% and is associated with fetal loss in almost half of cases [6,7]. With that said, there have been advocates to resect HCC at the earliest coupled with early termination of pregnancy [5,8]. Lesions that demonstrate specific hallmarks such as non-rim arterial enhancement and early portal venous washout, the diagnosis of HCC can then be made radiographically [9]. In our case, despite MRI findings, the patient does not have the risks factors, clinical signs of chronic liver disease and features of non-cirrhotic liver mass, the diagnosis of a benign liver lesion were predicated rather than classic HCC.

In pregnant patients with liver masses, the choice of imaging is limited as contrast-enhanced CT is rarely used to avoid exposing the fetus to ionizing radiation during gestation. Ultrasound by default is the most used modality due to no radiation risks in evaluating liver masses in pregnancy. It has a sensitivity of 90% in identifying liver tumors and can distinguish between cystic or solid lesions, single or multiple lesions but is unable to reliably differentiate between solid liver tumors [10]. Contrast agents used in MRI studies such as gadolinium contrast is controversial in pregnancy. Gadolinium is water-soluble and can cross the placenta into the fetal circulation and amniotic fluid. Free gadolinium is toxic thus it is usually administered in chelated (bound) form. In animal studies, gadolinium agents are teratogenic at high and repeated doses in their free form. The principal concern with gadolinium-based agents in humans is that the duration of fetal exposure is unknown due to the contrast present in the amniotic fluid that is swallowed by the fetus and reenters the fetal circulation. The longer gadolinium-based products remain in the amniotic fluid, the greater the potential for dissociation from the chelate and, thus, the risk of causing harm to the fetus [10]. The sensitivity (95%) and specificity (79%) of non-contrast MRI are comparable to those of contrast-enhanced MRI (sensitivity of 95% and specificity of 82%) [11]. Hence, non-contrast MRI can be used as a diagnostic tool when it is interpreted by a trained, and experienced radiologist.

Dealing with a pregnant mother is challenging as it involves both maternal and fetal wellbeing. The decision should not be made by a single practitioner but a collective decision in a multidisciplinary team of surgeons, oncologists, obstetricians, and radiologists to determine the appropriate timing for surgical intervention of the primary problem and at the same time evaluating the potential viability of the fetus throughout the pregnancy. In view of inconclusive MRI findings coupled with low-risk factors of HCC, we had decided to follow up her liver mass with imaging 6 weekly from the initial ultrasound scrutinizing the size of the tumor which remained static without invasion to surrounding structures and monitoring her liver function tests closely. However, considering the elevated serum tumor marker and inconclusive MRI with a LI-RADS 3 which translates to a possibility of 39% being malignant, a collective multidisciplinary decision was made to surgically intervene at 32 weeks of gestation. It is an option to wait and perform a delayed operation in the third trimester when the baby has higher survival outcomes. This is because at 28th gestational week is the critical point of fetal maturation due to fetal lungs achieving sufficient maturity as well as other organs. Performing a delivery before the 28th week of gestation results in less probable survivability of the baby. In addition, delivery before 32 weeks of gestation should be avoided because of fetus immaturity.

Though her final histopathology report revealed a grade 2 NET which is rare in liver as a primary site instead of a secondary deposit, her treatment was nevertheless approached in a similar fashion of NET treatment algorithm whereby ultimately a resection of the primary tumor was necessary. In fact, a quick search through PubMed reveals less than 10 reports of NETs occurring in pregnant women ie cervix and pancreas showing how rare these tumors are in pregnant patients [12], [13], [14]. NET can be further divided into functional and non-functional NET, in which in our case, it falls under the latter type as no features flushing or gastrointestinal related symptoms elicited. Hence, she did not require any somatostatin analogues. As 10%-14% well-differentiated NET of unknown primary site, it often presents initially with liver metastasis, hence patients must be actively worked up to look for its primary site with a gallium Ga-68 DOTATATE PET-CT scan [15]. Continuous follow up with yearly imaging is mandatory to ensure no recurrence of the tumor.

## Conclusion

Antenatal discovery of liver masses poses a diagnostic and management dilemma to clinicians. A multidisciplinary approach and collective decision making are crucial to determine the best approach tailored to the maternal and fetal benefit. In cases of inconclusive non-contrast MRI in pregnancy with low-risk factors and lack of clinical evidence of HCC, follow-up with imaging modalities aiming to intervene at the third trimester can offer safer, and promising outcomes.

## Patient consent

A written informed consent was obtained from the patient for the publication of this case report.
